# Circling Around the Uncanny Valley: Design Principles for Research Into the Relation Between Human Likeness and Eeriness

**DOI:** 10.1177/2041669516681309

**Published:** 2016-12-06

**Authors:** Stephanie Lay, Nicola Brace, Graham Pike, Frank Pollick

**Affiliations:** Department of Psychology, The Open University, Milton Keynes, UK; School of Psychology, University of Glasgow, Scotland

**Keywords:** uncanny valley, circularity, research methods, human likeness, eeriness

## Abstract

The uncanny valley effect (UVE) is a negative emotional response experienced when encountering entities that appear almost human. Research on the UVE typically investigates individual, or collections of, near human entities but may be prone to methodological circularity unless the properties that give rise to the emotional response are appropriately defined and quantified. In addition, many studies do not sufficiently control the variation in human likeness portrayed in stimulus images, meaning that the nature of stimuli that elicit the UVE is also not well defined or quantified. This article describes design criteria for UVE research to overcome the above problems by measuring three variables (human likeness, eeriness, and emotional response) and by using stimuli spanning the artificial to human continuum. These criteria allow results to be plotted and compared with the hypothesized uncanny valley curve and any effect observed can be quantified. The above criteria were applied to the methods used in a subset of existing UVE studies. Although many studies made use of some of the necessary measurements and controls, few used them all. The UVE is discussed in relation to this result and research methodology more broadly.

## Introduction

The idea that there is something odd about entities that fall into a uncanny valley (UV) between human and artificial has become a popular research area for disciplines such as robotic engineering, human-computer interaction, and psychology. This area of enquiry has progressed from its origins in 1970 as an untested thought experiment to become an established field which is developing ways of investigating the uncanny valley effect (UVE) and the perception of near human entities (NHEs). Research may address how we perceive humanity, and how we can improve designs for the appearance and behavior of artificial entities, and findings may develop our thinking about life in a future world where interactions with near-human and virtual entities will become commonplace. However, undertaking any research into the UV is more complex than it may initially appear. In particular, there is the potential for problems to arise in even establishing the existence of the UVE due to the use of circular methodology. This arises because of a tendency to see an entity as eliciting a UVE simply because it appears eerie, coupled with a tendency for an entity to be perceived as eerie simply because it is of near-human appearance. Research that subjectively selects potential UVE entities is, therefore, problematic, and instead, it may be important for research to consider definitions of characteristics such as human likeness and eeriness that can be more appropriately operationalized. The aim of the current article is to consider what methodological difficulties arise when studying the UVE, and how they might be overcome to produce research which is able to more objectively quantify and measure the effect.

## What Is the UVE?

The idea of the UVE originated in robot design and described an unusual pattern in how emotional responses to artificial entities changed with their increasing human likeness. As entities began to take on human characteristics, they initially seemed more appealing and likeable but this only held true up to a certain point, because when those characteristics became convincingly close to human, the entities suddenly seemed eerie and unsettling instead of more acceptable. This sudden dip in emotional response is the valley component of the term. It was deemed an UV because the responses to those NHEs were ones of unease or disquiet. The UVE was originally described by Mori (1970) who suggested that zombies, corpses, prosthetic hands, and Japanese Noh masks would fall into this valley. More recently, it has been given as a reason for why all sorts of nearly human-looking entities are perceived to be creepy.

Mori’s account of the UVE was translated from Japanese to English by MacDorman (2005) and depicted by using a graph with axes of human likeness and familiarity. Moving and still entities were plotted in separate curves. Human likeness ranged from the extremely artificial (an industrial robot) to completely human (a healthy living person). The original term used was *shinwakan,* a word relating to “familiarity,” “likableness,” “comfort level,” and “affinity.” In the translation, “familiarity” was the chosen term which proved complex to define, partly due to it having two meanings in English (an absence of novelty or a sense of closeness), leading to it being variously interpreted as meaning “positive affect,” “increasing affinity,” and “emotional warmth” (Ho & MacDorman, 2010). The curves described for moving and still entities both initially increase in familiarity as human likeness increases until the 60% to 65% human point where familiarity begins to decrease, finally reaching its lowest point at around 75% to 80% human. After this point, it rises steeply again until the highest familiarity is reached for a moving and healthy human being. The curves vary in magnitude according to whether the entities are still or moving, but on both there is a distinct dip in familiarity between 75% and 90% human where familiarity plummets, signaling that the entities are perceived as eerie, and this dip forms the UV.

When considering the precise nature of the UVE and its component dimensions, it is important to remember that Mori’s original article (Mori, 1970/2012) was written for Japanese robotics engineers and not intended as a scientific exploration of a psychological phenomenon. The nature of Mori’s original conceptualization of the UVE is demonstrated by two occasions when he revisited the theory after publishing the original paper. In 2005, he reflected on his decision to place corpses in the UV and suggested that when someone dies, their lack of animation can be unsettling, but if death released them from suffering or uncertainty then the stillness may also suggest that the person is now at peace, and this peaceful aspect may moderate any sense of uncanniness (Mori, 2005). He also suggested that it may have been wrong to position human beings as the highest point on the original curve, because idealized portrayals of the human form exist (e.g., in some Buddhist statues) which can appear more elegant, calm, and dignified than genuine humans. In an interview with Kageki (2012), Mori also suggested that the UVE may be caused by the viewer discovering a deception that the entity is not actually as human as it appears, and it is the discovery of a deceptively human appearance that causes eeriness when the familiarity drops away.

It is clear then that the origins of the UVE were not the result of scientific observation or experimentation, but more of an untested, theoretical construct aimed primarily at the aesthetics of robot design. As well as the problems of translating factors such as *shinwakan* that were mentioned earlier, problems also existed in the seemingly arbitrary placement of entities along the human likeness axes. For example, a stuffed animal was determined to be more human looking than was a humanoid robot, a distinction which is arguable and demonstrates that the formulation of the UV graph was not based on systematic, scientific categorization. Since Mori’s work was translated into English by MacDorman (2005), it has gained considerable attention, including in research that has attempted to identify and test the various elements involved to establish an empirical approach to the UVE, particularly in terms of reliably identifying what attributes of an entity might evoke a sense of eeriness.

## Why Is Circularity a Problem?

If it is possible to replicate the curve described by Mori (1970/2012) using experimental or observational methods, then this would provide empirical support for the UVE and be a baseline from which to explore its causes. One important point to bear in mind is that observing negative emotional reactions to certain entities is not by itself sufficient evidence for the existence of the UVE, and instead, it is necessary to demonstrate that the reaction to these entities fall within a “valley” when both the nature of the stimuli and the reaction are plotted on a graph using sufficiently calibrated measurements. Given the need for such calibration, it would seem reasonable that any study trying to show whether or not the UVE exists, or trying to explain the reasons behind it, should include measurement of human likeness, eeriness, and an emotional response. This emotional response may be the strange-familiar dimension depicted in Mori (1970/2012)’s graph, or more recent interpretations of affinity and warmth, such as Ho and MacDorman (2010).

One possible method of studying the UVE empirically is to collect examples of different NHEs and ask people how eerie they appear. However, there is a risk of methodological circularity in using such an approach, in that certain NHEs may be judged as eerie simply because they appear near-human rather than human or artificial (and hence likely to fall into the UV), and they are considered as examples of things that must belong to the UV because they look eerie. In conducting empirical studies of the UVE it is important to consider, therefore, how examples of NHEs are chosen, how the key characteristics of human likeness and familiarity are established, and how the emotional response to NHEs is defined and measured. If the examples are arbitrarily or subjectively selected, then although the findings may identify UV-like response patterns to those entities, it is not possible to draw conclusions about the existence of a UV; instead, it is only possible to conclude that such images appear to invoke a feeling of eeriness in those that encounter them. To provide evidence for the UVE, it is necessary to clearly and definitively position the NHEs in terms of their human likeness and familiarity, and quantify the emotional responses to the stimuli, in comparison to both more and less human like images.

## Design Principles for Researching the UVE

If the UVE exists, then it should be possible to replicate the graph Mori described using empirical data. Table 1 draws together a set of design principles, and a test that will assist such an endeavor. They are based a review of UVE studies conducted to date.

**Table 1.** Research Design Principles and Test for Investigating the UVE.

(1) Stimuli should cover a range of levels of human likeness, with a minimum of five points including human and nonhuman anchor points. It is not possible to draw conclusions about a continuum of human likeness when only two illustrative points are being compared or when the range does not include a human and nonhuman anchor. A full range would include 0% and 100% human, and 25%, 50%, and 75% human likeness.
(2) To produce the graph’s *X* axis, the human likeness of the stimuli displaying the identified quality should be controlled or measurable. Control would involve the selection of stimuli that vary in their human likeness in a systematic way (e.g., computer-generated entities or morphs between human and nonhuman) but if this is not possible, the stimuli should be independently rated to measure their human likeness.
(3) The *Y* axis of the graph, labeled as familiarity, represents the emotional response or reaction to the stimuli. This is not as clear-cut as the human likeness dimension as there are many different interpretations of what could be meant by this axis. Therefore, the emotion that is being measured should be defined and, to ensure psychological validity, response scales should be grounded in empirical evidence relating to human emotions.
(4) A rating of eeriness should be collected from participants for each of the stimuli in addition to the familiarity or emotional response measure. Without it, any observed valley could be explained as a mere dip in response to the stimuli, and it would not be possible to confidently assert that the valley was uncanny in its nature.
(5) If the principles earlier have been followed, it is possible to plot the two measurements of human likeness and emotional response against each other. To show a valley effect, the path described by the response measurement should display a single clear dip or deviation from a linear path, occurring somewhere between 50% and 100% on the human likeness axis. When this is considered against the ratings of eeriness, it should be possible to decide whether this represents an uncanny valley or not.

UVE = uncanny valley effect.

Items 1 and 2 are included to avoid the methodological circularity that arises when choosing an arbitrary selection of a small number of stimuli based on their near-human appearance, for example different types of androids or toys. A transition from nonhuman to human should be objectively quantified either by transforming a nonhuman gradually toward human or by ensuring that human likeness is rated independently from the other measurements. A minimum of five points is suggested because this is the smallest practical set that would include human and nonhuman anchors, and sufficient examples of NHEs that could feasibly allow a valley to be plotted. However, including stimuli at more than five points in that continuum would be preferable if the aim of the research is to precisely identify the location of the dip in the UV curve. Measuring eeriness in addition to familiarity (Items 5 and 3) allows the researcher to identify which stimuli were most closely associated with eeriness and then to explore these in more detail in further research to arrive at an understanding of the causes of the UVE. Types of emotional response that could be measured here include acceptability, warmth, pleasantness, and familiarity itself. Items 4 and 5 would allow researchers to see whether a pattern emerges that approximates the path described by Mori (1970/2012). This would mean that the stimuli falling into the valley would be those displaying high ratings of eeriness. Item 5 tests whether a valley can be described by examining whether there is a deviation from a linear trend in the relation between human likeness and emotional response. The ratings collected when Item 4 is used would allow a conclusion to be drawn whether it is uncanny or not.

## Reviewing the Principles Against UVE Research

There has been a considerable amount of research carried out into the UVE since 2005. (See Kätsyri, Förger, & Mäkäräinen, 2015, for a recent summary.) Studies were included in the present review if they were published between 2005 and 2016, collected primary data from participants, and had at least one research aim which included empirically testing whether the UVE existed, exploring why it might occur, or quantifying a relation between human likeness and eeriness. These studies have been categorized according to the type of research area they considered. Table 2 indicates which of the five principles were met for each study.

**Table 2.** Summary of Studies Detailing Whether the Design Criteria Were Met.

Research area	Study	1. Use of a range of stimuli varying in human likeness	2. Stimuli controlled or measured for human likeness	3. *Y* axis emotional response defined	4. Rating of eeriness collected	5. Deviation in response if 2 and 3 can be plotted	All criteria met?
Anomalous features	Green, MacDorman, Ho, and Vasudevan (2008)	Yes	Yes	Yes	Yes	Yes	Yes
	Hanson (2005)	Yes	Yes	Yes	Yes	No	No
	Seyama and Nagayama (2007)	Yes	Yes	Yes	No	Yes	No
Category boundaries and categorical perception	Burleigh, Schoenherr, and Lacroix (2013)	Yes	Yes	Yes	Yes	Yes	Yes
	Cheetham, Suter, and Jäncke (2011)	Yes	Yes	No	No	–	No
	Cheetham, Pavlovic, Jordan, Suter, and Jancke (2013)	Yes	Yes	No	No	–	No
	Cheetham, Suter, and Jancke (2014)	Yes	Yes	Yes	No	No	No
	Cheetham, Wu, Pauli, and Jancke (2015)	Yes	Yes	Yes	No	No	No
	Ferrey, Burleigh, and Fenske (2015)	Yes	Yes	Yes	No	Yes	No
	Matsuda, Okamoto, Ida, Okanoya, and Myowa-Yamakoshi (2012)	No	Yes	No	No	–	No
	Yamada, Kawabe, and Ihaya (2012)	Yes	Yes	Yes	No	Yes	No
Empathy and animacy	Gray and Wegner (2012)	No	No	Yes	Yes	–	No
	Looser and Wheatley (2010)	Yes	Yes	Yes	No	Yes	No
	Mathur and Reichling (2009)	Yes	No	Yes	No	–	No
	Mathur and Reichling (2015)	Yes	Yes	Yes	Yes	Yes	Yes
	McDonnell (2010)	No	No	Yes	No	–	No
	Woods (2006)	No	No	Yes	No	–	No
	MacDorman, Green, Ho, and Koch (2009)	Yes	Yes	Yes	Yes	Yes	Yes
Error sensitivity	Tinwell and Grimshaw (2009)	Yes	Yes	Yes	Yes	No	No
Evolutionary aesthetics	Lewkowicz and Ghazanfar (2011)	No	Yes	No	No	–	No
	Schneider, Wang, and Yang (2007)	No	Yes	Yes	No	Yes	No
	Steckenfinger and Ghazanfar (2009)	Yes^a^	Yes	No	No	–	No
Individual differences	Chaminade, Hodgkins, and Kawato (2007)	Yes	Yes	No	No	–	No
	Macdorman and Entezari (2015)	Yes	Yes	Yes	Yes	Yes	Yes
	Saygin, Chaminade, and Ishiguro (2010)	No	No	No	No	–	No
	Saygin, Chaminade, Ishiguro, Driver, and Frith (2012)	No	No	No	No	–	No
	Shimada, Minato, Itakura, and lshiguro (2007)	No	No	No	No	–	No
Perceptual mismatch	MacDorman and Chattopadhyay (2016)	Yes	Yes	Yes	Yes	Yes	Yes
	Piwek, McKay, and Pollick (2014)	Yes	Yes	Yes	No	Yes	No
	Seyama and Nagayama (2009)	Yes	Yes	No	No	–	No
	Thompson, Trafton, and McKnight (2011)	Yes	Yes	Yes	Yes	Yes	Yes
	Tinwell (2009)	Yes	Yes	Yes	No	No	No
	Tinwell, Nabi, and Charlton (2013)	Yes	Yes	Yes	Yes	Not reported	No

a No human faces were included in this study, so it is noted that the monkey faces acted as the equivalent to the human anchor.

It can be seen that 7 of these 33 studies met all five of the criteria that have been proposed here (Burleigh et al., 2013; Green et al., 2008; MacDorman & Chattopadhyay, 2016; MacDorman & Entezari, 2015; MacDorman et al., 2009; Mathur & Reichling, 2015; Thompson et al., 2011). These covered research into anomalous features, categorical perception, empathy, error sensitivity, and perceptual mismatch which supports Kätsyri et al.’s (2015) conclusion from a review of current research that perceptual mismatch theory provides good evidence for the existence of the UVE. These studies did not all find the valley deviation at the same position, and several found the most eerie stimuli were those at the midpoint of the human likeness continuum, rather than very close to the human endpoint as Mori (1970/2012) proposed. This suggests that if the valley exists it may not always occupy the region closest to human likeness.

While conducting this review, it became apparent that there is some inconsistency between the studies in their results and conclusions. This is the case for studies that have been categorized as applying the principles in their stimuli selection and measurement choices but that did not find a UVE but more so in the studies that have drawn conclusions about the UVE without all of those measures or controls in place. For example, Seyama and Nagayama (2007) concluded that infective processing mechanisms may be responsible for causing the UVE but without a measurement of eeriness in their studies it is hard to know if there was anything actually uncanny about the stimuli they tested. Studies that used a single android stimulus (e.g., Gray & Wegner, 2012) or which did not use human anchors for comparison (e.g., Woods, 2006) can provide useful suggestions as to what might cause the UVE but cannot provide evidence that these would apply to other androids or to entities which are nearly but not quite human in general. This highlights a difficulty in making comparisons between studies which have taken different approaches to the same problem of testing the UVE. It would be considerably easier to compare several studies if a consistent approach had been taken to measuring how human like and eerie their stimuli appeared to participants. Therefore, applying these principles in future research would help to build on those studies reported earlier that did meet all the criteria and found a UVE.

## Conclusions

The principles and test earlier were formulated as a design framework that could guide future research seeking to investigate the nature and causes of the UVE. By quantifying key variables, problems arising from methodological circularity can be avoided. The principles are not intended to be a prescriptive framework given the complexities involved in some of the approaches taken. For example, the field of category boundaries and categorical perception presents particular challenges where distinct measurements of perceptions of human likeness as defined here may overlap with the human likeness dimension measured in discrimination tasks (Cheetham et al., 2013). By comparing a range of research over several years and finding seven studies that met all the criteria and demonstrated an UVE, it has become apparent that these principles are certainly being applied by some researchers and so are not proposed as novel in their own right. Certainly, research in recent years does seem to have adopted these principles to good effect. However, these have not previously been drawn together as a framework of guiding principles to help as many studies as possible avoid the circularity problem. It is hoped that in setting out these principles and giving examples of how they have and have not been applied, this article will assist those seeking to contribute to a fuller understanding of the UVE, and explanations for why, and how, it can evoke its characteristic and unsettling response.
